# Acute effects of virtual reality-based relaxation and exergaming on primary dysmenorrhea symptoms

**DOI:** 10.55730/1300-0144.5981

**Published:** 2025-02-27

**Authors:** Muhammed Şeref YILDIRIM, Merve ÇOĞ, Büşra Mehder AKBAŞ, Sinem SALAR, Hilal KEKLİCEK

**Affiliations:** 1 Department of Physiotherapy and Rehabilitation, Faculty of Health Sciences, Trakya University, Edirne, Turkiye; 2 Department of Occupational Therapy, Faculty of Health Sciences, Trakya University, Edirne, Turkiye

**Keywords:** Exercise therapy, pain management, primary dysmenorrhea, progressive muscle relaxation, virtual reality

## Abstract

**Background/aim:**

Primary dysmenorrhea (PD) is a menstrual disorder with significant physical and psychological impacts. Physical activity and relaxation techniques are proven methods for managing PD. Advances in virtual reality (VR) suggest that immersive relaxation and exergaming could provide new avenues for symptom relief. This study aimed to investigate the acute effects of VR-based interventions on PD symptoms and compare them with a control group receiving Jacobson’s Relaxation

**Materials and methods:**

A randomized, controlled, single-blind trial was conducted with 43 female participants aged 18–30 years with regular menstrual cycles and PD. Participants were allocated to three groups: (I) immersive VR relaxation, (II) nonimmersive VR exergaming, or (III) Jacobson’s relaxation (control group). Each participant completed a single 20-min session of their assigned intervention. Primary outcomes included abdomino-pelvic pain intensity, menstrual symptom severity, sleep quality, and perceived intervention effectiveness.

**Results:**

All interventions reduced pain (r = 0.78–0.85) and symptom severity (r = 0.73–0.88) (p < 0.05), with no sleep quality changes. Between-group comparisons showed the control group to be more effective than nonimmersive VR exergaming in reducing menstrual symptom severity (p = 0.021, η^2^ = 0.144) and yielded higher perceived effectiveness (p = 0.010, η^2^ = 0.182).

**Conclusion:**

Both VR-based interventions and the control group effectively alleviated PD symptoms, with the control group providing the most consistent symptom relief. Despite promising results for VR-based methods, traditional relaxation may be more accessible and better tolerated.

## 1. Introduction

Primary dysmenorrhea (PD) is characterized by severe pain in the suprapubic and abdominal regions during menstruation, without any identifiable pelvic pathology [1]. The prevalence of PD ranges from 45% to 72% in reproductive-age women, escalating to 93% in adolescents [1]. During menstruation, abdomino-pelvic pain may be accompanied by additional symptoms such as bloating, headache, nausea, fatigue, and sleep disturbances, typically lasting 24–48 h [2, 3]. Research indicates that these symptoms can negatively affect social and sports activities, as well as academic and work performance [4, 5].

Various multimodal approaches are employed to alleviate PD symptoms, including relaxation techniques, massage, physical activity, hot/cold compresses, and electrotherapy [6–9]. Physical activities like aerobic exercise, yoga, and isometric exercise reduce stress, improve mood, alleviate pain, and decrease prostaglandin levels, easing uterine cramps [10]. Progressive relaxation techniques, on the other hand, decrease sympathetic activity, reducing stress and anxiety. Additionally, the increased blood flow resulting from peripheral vasodilation helps to reduce menstrual cramps and pain perception [11–13]. The effectiveness of traditional physical activity and relaxation techniques in improving PD symptoms has been confirmed by numerous studies [6, 9, 14–16].

The proven efficacy of traditional methods in managing PD symptoms can be further enhanced with the rapidly advancing VR technology. Immersive virtual reality (IVR), which isolates the user from their real environment and creates a strong sense of presence in a safe, virtual space, can offer a significant advantage over traditional relaxation techniques. The immersive experiences in virtual environments amplify the distraction effect, a cognitive-behavioral pain management strategy [17]. This allows individuals to better engage in relaxation processes, thereby improving pain management [18]. With only a single study utilizing IVR relaxation for menstrual symptom management, Heydarpour and Dehghan’s research [19] offers valuable insight. They found IVR to be as effective as hypnotherapy in reducing pain, underscoring VR’s potential as an alternative for managing menstrual symptoms through immersive engagement.

Similarly, integrating technology into physical activity can also support PD symptom management. Exergaming, for instance, provides intense visual, auditory, and sensory stimuli during physical activity, diverting users’ attention from internal cues like fatigue, increased heart rate, and pain. This enriched experience boosts motivation, encouraging users to participate in exercise for longer durations and with greater effectiveness [20]. Although there is no research on exergaming’s impact on menstrual pain, studies indicate that exergaming can significantly reduce other types of pain, such as chronic musculoskeletal, thermal, and phantom limb pain, highlighting its potential in diverse pain management contexts [21]. Moreover, research indicates that participants report higher enjoyment, interest, and intrinsic motivation during exergaming compared to traditional exercise [22, 23]. These findings suggest that exergaming may contribute to pain and stress reduction in PD through effective physical exercise participation and the creation of a positive emotional state.

There is a lack of research comparing the effectiveness of different VR interventions with traditional methods in managing dysmenorrhea symptoms, particularly regarding their advantages and limitations. In this study, Jacobson’s Relaxation was chosen as the control group due to its well-documented efficacy in reducing menstrual pain and its widespread acceptance as a standard relaxation method [9]. Our study aims to investigate the acute effects of Immersive Virtual Reality Relaxation (IVR-R) and Nonimmersive Virtual Reality Exergaming (NIVR-EXE) on PD symptoms and compare these effects with a control group receiving Jacobson’s Relaxation (CON-JR), a traditional relaxation method.

## 2. Materials and methods

### 2.1. Participants

The research received approval from the Trakya University Faculty of Medicine Clinical Research Ethics Committee (decision 5/29, dated 02.27.2023), prior to recruiting the first participant. It was conducted in accordance with the ethical principles of the Declaration of Helsinki and carried out at the Trakya University Faculty of Health Sciences Exercise Laboratory. Data collection was conducted between May 2023 and February 2024.

**Figure 1:**
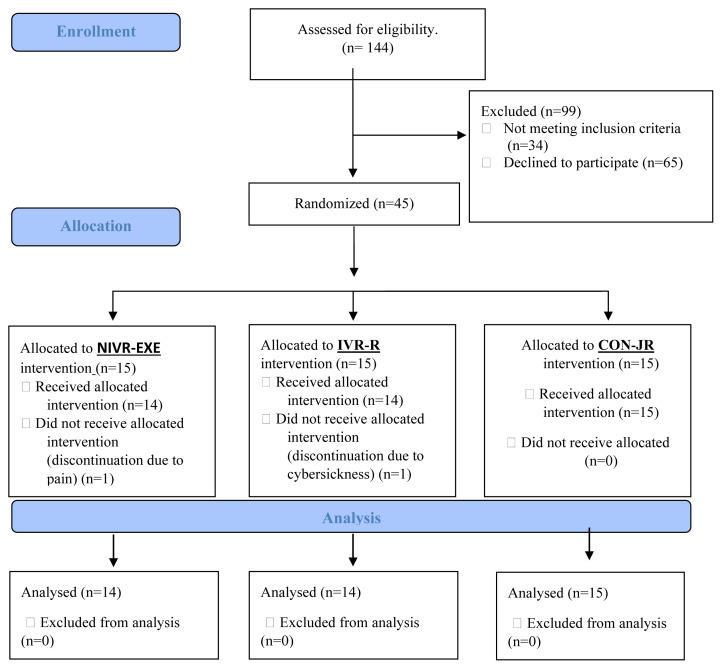
CONSORT flow diagram illustrating the participants in the study. IVR-R: Immersive virtual reality relaxation, NIVR-EXE: Nonimmersive Virtual Reality Exergaming, CON-JR: Control group using Jacobson’s Relaxation.

We invited healthy females from the university community who were contacted by social media announcements to take part in our study at the Department of Physiotherapy and Rehabilitation, Faculty of Health Sciences, Trakya University. Inclusion criteria required (1) women aged 18–30 with no pregnancy history (2) a regular menstrual cycle (28 ± 7 days) over the past 6 months, (3) absence of any chronic medical condition to rule out potential confounding factors, and (4) menstrual pain with a Visual Analog Scale score >4 during this period [24]. Exclusion criteria were: (1) regular medication use or medical treatment during the study (e.g., oral contraceptives, antidepressants); (2) Recent musculoskeletal trauma or surgery, or chronic musculoskeletal disorders affecting pain perception (e.g., fibromyalgia, pelvic floor dysfunction); (3) Intrauterine device (IUD) use or secondary dysmenorrhea-related conditions (e.g., endometriosis, fibroids, ovarian cysts),; and (4) consumption of painkillers, alcohol, recreational drugs, or similar substances within the last 48 h. All inclusion and exclusion criteria were verified through participants’ national health records (E-Nabız system). Participant flow through the study is illustrated in the CONSORT diagram (Figure 1).

### 2.2. Experimental procedures

This study was randomized and single-blinded. The assessors were blinded to the study groups. Participants were randomly assigned to one of three groups using online software (https://www.randomizer.org).

**Figure 2:**
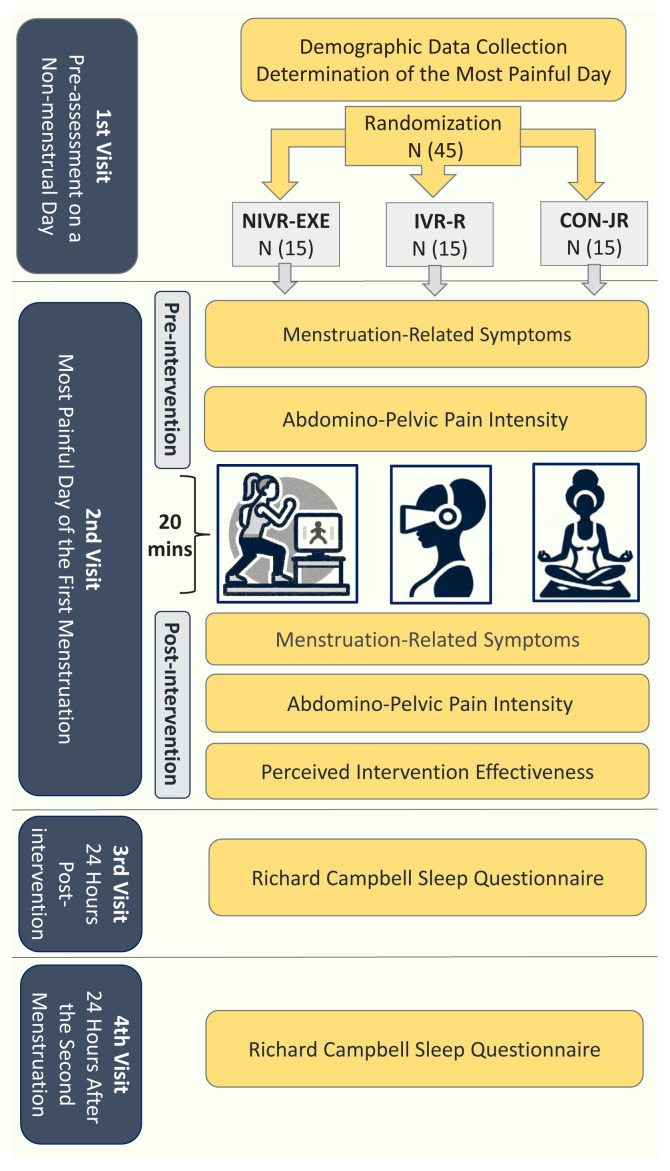
Overview of experimental procedures. NIVR-EXE: Nonimmersive Virtual Reality Exergaming, IVR-R: Immersive Virtual Reality Relaxation, CON-JR: Control Group-Jacobson’s Relaxation.

The participants visited our laboratory four times within the scope of the study. Figure 2 shows an overview of the experimental procedures.

Visit 1: Preassessment on a nonmenstrual day

Volunteers who expressed interest in participating in the study visited the laboratory on a nonmenstruating day when they were feeling well. Their eligibility was assessed, and those meeting the study criteria provided written informed consent. Demographic data, including age, height, weight, and body mass index (BMI), were collected. To determine the most painful day of their menstrual cycles, participants marked their pain levels on five separate Visual Analog Scales (VAS) for the day before bleeding and days 1, 2, 3, and 4 of bleeding. Participants were then scheduled for their second visit on the most painful day of their first menstrual cycle following enrollment.

Visit 2: Most painful day of the first menstruation

Baseline assessments were conducted to evaluate pre-intervention menstruation-related symptoms and Abdomino-Pelvic Pain Intensity (APPI). After these assessments, participants completed their assigned 20-min interventions:

Immersive virtual reality relaxation (IVR-R): The IVR-R group experienced a 20-min immersive relaxation session using the HTC Vive Pro virtual reality system and the “Nature Treks” application, while seated comfortably. The HTC Vive Pro, equipped with high-resolution visuals, a 360-°field of view, and over-ear headphones that provide audio isolation, offers a heightened sense of presence, effectively transporting users to calmer environments [25]. Nature Treks allows for free exploration of various serene natural settings, accompanied by soothing soundscapes, promoting a sense of peace and tranquility [26]. Participants were instructed to select the natural environment they found most relaxing from options such as “Blue Ocean”, “Orange Sunset”,”Violet Down”, “White Winter”, and “Green Meadows”, and spent 20 min immersed in their chosen virtual environment.

Nonimmersive Virtual Reality Exergaming (NIVR-EXE): Participants in the NIVR-EXE group engaged in a 20-min calisthenic exercise session guided by the Kinect Adventures game on the Xbox Kinect 360 system. This nonimmersive VR technology utilizes an infrared camera sensor to track full-body movements in real-time, seamlessly translating them into the game environment [27]. The exercise session consisted of playing two rounds each of the game segments “20,000 Leaks”, “Reflex Ridge”, and “River Rush”, each lasting approximately 3 min [28].

Control Group - Jacobson’s Relaxation (CON-JR): This group underwent 20 min of progressive relaxation training as developed by Dr. Jacobson [29]. The training involves a series of deep diaphragmatic breaths followed by the systematic tensing and relaxing of 16 major muscle groups throughout the body. Participants were guided through the exercise by an approximately 20-min audio recording.

Following the assigned activities, Perceived Intervention Effectiveness (PIE), post-intervention menstruation-related symptoms, and APPI were measured.

Visit 3: 24 ± 2 h postintervention

Participants made their third visit to the laboratory 24 ± 2 h after the intervention. During this visit, the six-item Richard Campbell Sleep Questionnaire (RCSQ) was used to score sleep quality on the intervention night.

Visit 4: Follow-up assessment

The final visit to the laboratory occurred approximately one month after the intervention day, 24 h after the most painful day of the subsequent menstrual cycle. During this visit, RCSQ scores on the nonintervention night were evaluated.

### 2.3. Outcome measures

Abdomino-pelvic pain intensity (APPI):

VAS was used to measure abdomino-pelvic pain intensity. A 10 cm line was labelled with “0 cm” indicating no pain and “10 cm” representing unbearable pain at each end. Participants were asked to indicate their pain level by either drawing a line, placing a dot, or marking a point along the scale [30]. Pain assessments were made for the most painful menstrual day and abdomino-pelvic pain on the intervention day.

Menstruation-related symptoms:

The Daily Symptom Rating Scale (DSRS), a 17-item self-report tool, was used to assess the intensity of various menstrual symptoms, including pain, mood swings, and physical discomfort. Each symptom was rated on a scale from 0 (no symptoms) to 5 (severe), with higher scores indicating greater symptom severity. The Turkish version of the scale has been validated and shown to be reliable [31].

Perceived intervention effectiveness (PIE):

VAS is a simple and widely used tool, commonly recognized for assessing pain but also applied to measure other subjective experiences, including depression, cough severity, disease activity, and fatigue [32–34]. In our study, VAS was employed to assess participants’ perceived effectiveness of the intervention in managing menstrual symptoms. After the intervention, participants rated how effective it was in helping them cope with menstrual symptoms and how they felt overall on a 0–10 scale, with 0 indicating no effect and 10 indicating maximum effectiveness.

Sleep quality:

RCSQ was used to assess sleep quality on the night following the intervention and during a nonintervention menstruation day. The six-item self-report questionnaire was validated in Turkish, with higher scores indicating better sleep quality [35].

### 2.4. Data analysis

Statistical analyses were performed using the Statistical Package for Social Sciences version 26.0 (SPSS Inc., Chicago, IL, USA). The significance level (α) was set at 0.05. The normality of the data distribution was assessed using visual methods (histograms, probability plots) and the Shapiro-Wilk test. Within-group comparisons of RCSQ scores between intervention and nonintervention nights, PIE scores, and pre and postintervention APPI and DSRS scores were conducted using the Wilcoxon Signed Rank test. The Kruskal-Wallis test was used to compare each questionnaire outcome between groups. In cases where the Kruskal-Wallis test revealed significant differences between groups for PIE and postintervention DSRS scores, pairwise comparisons with Bonferroni correction were employed to identify the specific groups responsible for these differences. To assess the clinical relevance of the findings, a posthoc power analysis was performed. Based on postintervention APPI levels (VAS), the effect size was calculated using Cohen’s f, yielding a power of 93% and an effect size of 0.60.

## 3. Results

The study included 43 volunteer women with ages ranging from 19 to 25 (21.17 ± 1.07). The groups exhibited no significant differences in age, height, body weight, or body mass index (BMI) (p > 0.05, Table 1).

In the pre-assessment, 34 women (79.1%) reported the first day of bleeding as the most painful day of menstruation, while 9 women (20.9%) reported the second day.

Within-group comparisons revealed a significant decrease in APPI and DSRS scores after the intervention in all groups, while RCSQ scores remained unchanged across all groups

In the pre-intervention state, between-group comparisons showed that the randomized groups were similar in terms of pain (pre-intervention APPI scores) and menstrual symptom severity (preintervention DSRS scores). After the intervention, the similarity in pain levels (postintervention APPI scores) between groups was maintained. However, a significant difference was observed in symptom severity (postintervention DSRS scores) between at least one of the intervention groups (Table 2).

No significant difference in sleep quality was observed across all groups, either on the intervention or nonintervention nights. However, there was a significant difference between the intervention groups in terms of PIE scores, reflecting participants’ perceived effectiveness of the interventions (Table 2).

Posthoc analyses revealed that the between-group differences in postintervention DSRS scores and PIE scores were in favour of the CON-JR group, primarily due to the difference between the NIVR-EXE and CON-JR groups (Tables 2–3).

## 4. Discussion

This study investigated the acute effects of virtual reality-based relaxation and exercise methods on PD symptoms and compared these methods with a traditional relaxation technique, Jacobson’s Relaxation. Within-group analyses showed that all three interventions resulted in beneficial effects, with reductions in APPI and DSRS scores observed across all groups. However, no change was observed in RCSQ scores. In the between-group analyses conducted to compare the effects of the three interventions, it was found that the CON-JR group was superior to the NIVR-EXE group in terms of DSRS and IEAS scores.

The within-group reductions in APPI and DSRS scores observed in the CON-JR group are consistent with previous research findings that have repeatedly demonstrated the effectiveness of traditional relaxation techniques in improving PD symptoms [6, 14, 15]. On the other hand, within-group reductions in the IVR-R group are consistent with a previous study by Heydarpour and Dehghan [19], the only known study to date investigating VR-based relaxation for menstrual symptom management. This alignment suggests that immersive VR relaxation could be a viable alternative to traditional methods to symptom relief. One of the significant findings of our study is that, to the best of our knowledge, it is the first to demonstrate the effectiveness of nonimmersive VR-based exergaming (NIVR-EXE) in alleviating PD symptoms. This finding is particularly important, as while previous research has shown that moderate physical activity can relieve menstrual symptoms [22, 23], many women choose not to engage in exercise during their menstrual period due to a combination of physical discomfort and psychological factors [36]. In our study, with the exception of one participant, all women in the exergaming group successfully completed the 20-min session without reporting any discomfort. This suggests that exergaming may help distract participants from discomfort and menstrual pain, encouraging sustained participation in physical activity and potentially alleviating PD symptoms. This finding is consistent with McDonough et al. [37], who reported that exergaming promotes higher enjoyment and lower perceived exertion compared to traditional exercise in college students. Such features may have contributed to the successful completion of the exercise session by participants in the NIVR-EXE group despite menstrual discomfort

Beyond pain and symptom relief, sleep quality was evaluated using RCSQ scores on both the intervention and nonintervention nights, revealing no significant differences across any of the groups. Contrary to our expectations, this suggests that the interventions did not notably impact sleep quality during menstruation. This finding contrasts with previous studies that reported improved sleep quality following progressive muscle relaxation during the menstrual phase [15] and postmenopausal period [38], as well as research indicating that physical activity can alleviate menstrual symptoms [22, 23]. A possible explanation for this discrepancy is that our interventions were limited to a single session, whereas prior studies demonstrated effects over longer-term applications. Additionally, our reliance on the subjective RCSQ, rather than objective assessments like polysomnography or actigraphy, may have contributed to the null effect observed in sleep quality outcomes.

In addition to these findings, the between-group comparisons revealed the homogeneity of the groups across all outcome measures prior to the interventions. Post-intervention, the APPI scores showed that the interventions were similarly effective in reducing pain across all groups. However, when examining the more comprehensive DSRS scores which assess not only pain but also mood swings, fatigue, and emotional responses the CON-JR and IVR-R groups showed similar efficacy, with CON-JR proving more effective than NIVR-EXE. Similarly, the post-intervention PIE scores also showed a significant difference in favor of the CON-JR group. This finding aligns with Chen & Li [39], who found that while both VR and meditation reduced heart rate after stress induction in healthy young adults, traditional meditation was superior in enhancing alpha brainwave activity, indicating a deeper relaxation response. These findings, together with ours, suggest that although VR can promote relaxation, traditional techniques like Jacobson’s relaxation may lead to a deeper physiological state of calm, possibly due to cognitive familiarity and focused mental engagement. Taking into account these findings and the participants who dropped out from the IVR-R (cybersickness) and the NIVR-EXE group (pain), it appears that participants felt better overall during the traditional Jacobson’s relaxation and experienced more effective symptom relief compared to the other methods. The familiarity of traditional relaxation methods may have contributed to the superior outcomes in the CON-JR group, while the VR methods’ unfamiliarity may have affected their efficacy. Given that our interventions were applied in a single session, regular practice could yield different results.

### 4.1. Limitations

This study has some limitations. First, sleep quality was assessed with the self-reported RCSQ, which may lack the precision of objective tools like polysomnography. Second, the interventions were conducted only once, potentially limiting the observed effects on symptoms and sleep; repeated sessions could yield more consistent results. Lastly, participants unfamiliar with VR-based interventions may have experienced discomfort. Pre-screening for cybersickness in the IVR-R group could have helped identify and exclude those at higher risk, improving overall comfort and intervention effectiveness.

## 5. Conclusion and recommendations

Our study demonstrates that both VR-based relaxation and exergaming methods offer promising benefits for alleviating PD symptoms. However, participants reported feeling most comfortable and experiencing the most effective symptom relief with Jacobson’s Relaxation, suggesting a preference for traditional relaxation methods. Despite the benefits of VR-based interventions, individualized assessments should be prioritized, especially given the potential for adverse effects in certain participants. For instance, in nonimmersive VR-based exergaming, modifying exercise intensity with gentler activities such as stretching or light cardio, or incorporating seated upper-extremity exercises may better suit individuals who struggle to tolerate higher physical intensity during their menstrual period. To mitigate the risk of cybersickness, especially in immersive VR relaxation, using tools like the Cybersickness Questionnaire for VR (CSQ-VR) [40] following brief initial VR sessions can help identify susceptible participants early on.

Future studies may explore the long-term application of these interventions to assess their potential effects on menstrual symptoms and sleep quality. These findings contribute to a growing body of evidence on VR’s potential in symptom management, underscoring the need for tailored approaches to optimize both comfort and effectiveness for individuals with PD.

## Figures and Tables

**Table 1:** Demographic characteristics of the participants.

	NIVR-EXE n = 14	IVR-R n = 14	CON-JR n = 15	p
	Mean ± SD	Mean ± SD	Mean ± SD	
Age (year)	21.06 ± 1.57	21.06 ± 0.79	21.4 ± 0.63	0.293
Height (cm)	162.66±6.93	166.26±1.51	161.13 ± 5.39	0.063
Body mass (kg)	63.93 ± 8.87	60.2 ±13.44	58.13 ± 10.31	0.077
BMI (kg/m^2^)	24.10 ± 3.50	21.60 ±3.82	21.97 ± 4.98	0.054

NIVR-EXE: Nonimmersive Virtual Reality Exergaming), IVR-R: Immersive Virtual Reality Relaxation), CON-JR: Control group receiving Jacobson’s Relaxation, SD: Standard Deviation, p^*^ < 0.05

**Table 2:** Within-group and between-group comparisons of research parameters before and after the intervention.

	NIVR-EXE n = 14	IVR-R n = 14	CON-JR n = 15	Kruskall-Wallis
	Mean ± SD	Mean ± SD	Mean ± SD	p/effect size (η^2^)
Preintervention APPI	6.43 ± 1.40	6.80 ± 1.72	7.40 ± 1.96	0.054
Postintervention APPI	4.25 ± 2.14	3.36 ± 1.90	2.8 ± 1.36	0.195
Wilcoxon p/effect size (r) (APPI)	**0.003** ^*^ **/0.78**	**0.002** ^*^ **/0.83**	**p < 0.001** ^*^ **/0.85**	
Preintervention DSRS	33.50 ± 10.94	35.42 ± 8.44	37.40 ± 11.11	0.389
Postintervention DSRS	16.64 ± 13.84	10.78 ± 13.58	3.86 ± 6.51	**0.021** ^*^ **/0.144**
Wilcoxon p/effect size (r) (DSRS)	**0.006** ^*^ **/0.73**	**0.002** ^*^ **/0.85**	**0.001** ^*^ **/0.88**	
RCSQ (intervention night)	354.28 ± 77.03	368.57 ± 82.07	370.66 ± 80.21	0.935
RCSQ (nonintervention night)	320.00 ± 85.12	305.71 ± 90.01	338.67 ± 82.53	0.717
Wilcoxon p/effect size (r) (RCSQ)	0.064	0.116	0.258	
PIE score	6.71 ± 1.76	7.21 ± 1.67	7.93 ± 1.79	**0.010** ^*^ **/0.182**

NIVR-EXE: Nonimmersive Virtual Reality Exergaming; IVR-R: Immersive Virtual Reality Relaxation; CON-JR: Control group receiving Jacobson’s Relaxation; APPI: Abdomino-Pelvic Pain Intensity; DSRS: Daily Symptom Rating Scale (Menstruation-Related Symptoms); PIE: Perceived Intervention Effectiveness; RCSQ: Richard Campbell Sleep Questionnaire; SD: Standard Deviation. Effect sizes: η^2^ for Kruskal-Wallis test (0.01 = small, 0.06 = medium, 0.14 = large); r for Wilcoxon Signed Rank test (0.1 = small, 0.3 = medium, 0.5 = large). Significant results were reported with p^*^ < 0.05.

**Table 3:** Posthoc analysis of the differences in postintervention DSRS and PIE scores.

Posthoc comparisons	Postintervention DSRS	PIE
	p/effect size (r)	p/effect size (r)
CON-JR vs. NIVR-EXE	**0.006** ^*^ **/0.57**	**0.008** ^*^ **/0.53**
CON-JR vs. IVR-R	0.266	0.157
IVR-R vs. NIVR-EXE	0.102	0.904

NIVR-EXE: Nonimmersive Virtual Reality Exergaming; IVR-R: Immersive Virtual Reality Relaxation; CON-JR: Control group receiving Jacobson’s Relaxation; DMSSS: Daily Menstrual Symptom Rating Scale; IEAS: Intervention Effectiveness Assessment Scale. Effect sizes (r): 0.1 = small, 0.3 = medium, 0.5 = large. Significant comparisons were evaluated with Bonferroni correction, p < 0.017^*^.
